# Pure Large Nested Variant of Urothelial Carcinoma (LNUC) Is the Prototype of an *FGFR3* Mutated Aggressive Urothelial Carcinoma with Luminal-Papillary Phenotype

**DOI:** 10.3390/cancers12030763

**Published:** 2020-03-24

**Authors:** Veronika Weyerer, Markus Eckstein, Eva Compérat, Hendrik Juette, Nadine T. Gaisa, Yves Allory, Robert Stöhr, Bernd Wullich, Morgan Rouprêt, Arndt Hartmann, Simone Bertz

**Affiliations:** 1 Institute of Pathology, University Hospital Erlangen, Friedrich-Alexander-Universität Erlangen-Nürnberg, 91054 Erlangen, Germany; veronika.weyerer@uk-erlangen.de (V.W.); markus.eckstein@uk-erlangen.de (M.E.); robert.stoehr@uk-erlangen.de (R.S.); Arndt.Hartmann@uk-erlangen.de (A.H.); 2 Hôpital Tenon, HUEP, Sorbonne University, 75020 Paris, France; eva.comperat@aphp.fr; 3 Institute of Pathology, Ruhr-University Bochum, 44789 Bochum, Germany; hendrik.juette@ruhr-uni-bochum.de; 4 Institute of Pathology, RWTH University Aachen, 52074 Aachen, Germany; ngaisa@ukaachen.de; 5 Department of Pathology, Hôpital Foch, Université Versailles-Saint-Quentin-en-Yvelines, Université Paris-Saclay, 92150 Suresnes, France; Yves.Allory@curie.fr; 6 Institut Curie, 75248 Paris, France; 7 Department of Urology and Pediatric Urology, University Hospital Erlangen, Friedrich-Alexander-Universität Erlangen-Nürnberg, 91054 Erlangen, Germany; Bernd.Wullich@uk-erlangen.de; 8 Sorbonne Université, GRC n5, ONCOTYPE-URO, AP-HP, Urology, Hôpital Pitié-Salpêtrière, 75013 Paris, France; mroupret@gmail.com

**Keywords:** urothelial carcinoma, large nested variant, LNUC, *FGFR3* mutation, luminal subtype

## Abstract

Since 2016, large nested urothelial carcinoma (LNUC) has been included within the WHO classification of urothelial tumors. Limited reports with mainly small case series have confirmed the malignant behavior of LNUC despite its bland morphological appearance. We evaluated, for the first time, markers for new immunooncological or targeted therapies including *FGFR3* mutational status and PD-L1 status, the frequency of *TERT*-promoter mutations and the molecular subtype in a cohort of 25 LNUC using SNaPshot analysis and immunohistochemistry. Of the 25 cases, 17 were pure LNUC, with 13 showing an additional exophytic papillary/papillary-like component. Seven mixed LNUCs presented areas of classical nested variant urothelial carcinoma (NVUC) and one showed a component of conventional urothelial carcinoma. Of the 17 evaluable pure LNUCs, 16 were *FGFR3*-mutated with identical mutations in their concomitant papillary/papillary-like components. An *FGFR3* mutation was found in 1/7 evaluable mixed LNUCs combined with NVUC. *TERT*-promoter mutations were detected in 86.7% pure and 83.3% mixed tumors. Immunohistochemistry revealed a luminal phenotype; PD-L1 was negative in the majority of tumor cells and tumor-associated immune cells. Pure LNUC is a prime example of a luminal, *FGFR3*-mutated, mostly PD-L1-negative tumor. In contrast, *FGFR3* mutations seem to be rare in mixed LNUC, which may indicate a different pathway of tumor development.

## 1. Introduction

Urothelial bladder cancer presents as urothelial carcinoma (UC) in 80%–90% of patients; the remaining patients present with non-urothelial tumors. Apart from squamous and glandular differentiation, variants with distinctive histomorphological patterns have been defined, which can be found in up to 33% of radical cystectomies as pure variant tumors or mixed tumors with conventional urothelial carcinoma (cUC) or other variants [1]. However, the actual prevalence of variant morphologies is not completely clear, since they may be under-recognized. The biological background, and therefore implications for clinical management, of reported variants of UC are not yet well understood and are still under investigation [2]. The large nested variant of urothelial carcinoma (LNUC) was first described in 2011 by Cox and Epstein [3] and has only recently been included in the 2016 World Health Organization (WHO) Classification system within the nested variant of urothelial carcinoma (NVUC) [4]. Morphologically, LNUC usually presents with large-sized well-delineated or irregular tumor nests with a bland cytology invading the detrusor muscle [3]. The growth pattern of LNUC is similar to the nested variant of urothelial carcinoma, with tumor nests lacking inflammatory and/or desmoplastic stroma reaction. This was probably the reason for combining LNUC and NVUC into one group in the WHO classification. Since the first description, only two clinicopathological studies demonstrated the aggressive behavior of this specific variant [5,6]. However, to date, no molecular data on LNUC have been available. 

Until recently, platin-based chemotherapy regimens were the gold standard in the therapy of patients with muscle-invasive bladder cancer (MIBC). Advances in the therapeutic management of invasive UC include immunooncological therapies with PD-1/PD-L1 inhibitors, as well as targeted therapies with *FGFR* inhibitors. Medications from both groups have been approved by the FDA (https://www.fda.gov/drugs/development-approval-process-drugs/drug-approvals-and-databases) and are currently being tested in clinical trials [7]. Moreover, molecular subtypes of UC based on gene expression analyses are supposed to have predictive value [8]. A molecular taxonomy consensus classification of UC summarizing the results of several gene expression studies revealed six bladder cancer subtypes [9].

In the present study, we evaluated *FGFR3* mutational status, PD-L1 tumor cell and immune cell expression and the molecular subtype in a cohort of 25 LNUCs.

## 2. Results

### 2.1. Clinical Data and Histomorphological Evaluation

**Figure 1:**
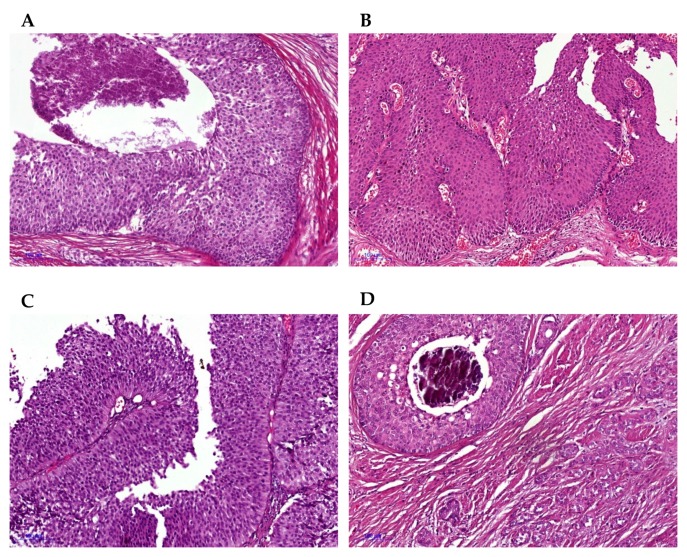
(**A**) Large nested urothelial carcinoma: typical histomorphology showing large-sized well delineated nests with bland cytology infiltrating the detrusor muscle; (**B**) inverted growth pattern in LNUC; (**C**) Papillary-like exophytic component; (**D**) LNUC combined with classical nested variant urothelial carcinoma (NVUC) (all H&E; all 100 fold original magnification).

Within our cohort of 25 patients diagnosed with LNUC, 18 were male, four were female, and three were not known. Twenty-four of the 25 tumors within the cohort were MIBC (≥pT2) and high-grade tumors according to the WHO classification (Table 1). In one case, we did not see tumor infiltration of the detrusor muscle, however, in this case we received tumor tissue from an osseous metastasis. Histomorphologically, LNUC showed medium to large-sized nests with a predominantly bland cytological appearance, with low mitotic activity invading the detrusor muscle and frequent central comedo-like necrosis. There was only very limited stromal response with, at most, sparse immune cell infiltration and little to a complete absence of stromal desmoplasia. In addition, 12/25 cases presented with a papillary and/or inverted papillary-like carcinoma component, giving the impression of an exophytic and partially inverted UC. However, compared to conventional non-invasive papillary UC, the papillary structures of LNUC frequently were much more plump, elongated and rarely branched. Of the 25 cases, 17 were pure LNUC; the remaining cases (8/25) presented with a mixed morphology combined with the classical nested variant with small-sized nests (n = 7) or cUC (n = 1). Other rare variant morphologies were not detected. Figure 1 demonstrates the histomorphological characteristics and phenotypes of LNUC.

### 2.2. Mutation Analysis 

Seventeen of 23 (73.9%) evaluable cases were *FGFR3* mutated, 16 of which were pure LNUC. The only mixed LNUC with a *FGFR3* mutation was an LNUC combined with NVUC. In detail, a p.S249C *FGFR3* mutation was found in eight (47.1%), p.Y375C in six (35.3%) and p.R248C in three (17.6%) cases; the mixed LNUC case had a p.S249C mutation. The *FGFR3* mutations identified in the muscle-invasive component of pure LNUC matched with the mutations in their papillary-like components in all evaluable cases. The distribution of *FGFR3* mutations within the tumor components is shown in Table 2. 

Eighteen of 21 (85.7%) successfully investigated cases showed one of the hotspot promoter mutations of the *TERT* gene. Twelve cases presented the -124 G>A mutation, four cases -146 G>A, and two cases the -57A>C transition (Table 3). One mixed and one pure case showed discordant results with wild type mutational status in LNUC and a mutation in the nested or papillary-like component. One case showed concomitant mutations of -124 G>A and -146 G>A within the papillary-like component and -124 G>A only in the LNUC component. In the remaining six cases, *TERT* mutations matched with the mutations in other tumor components. *TERT*-promoter mutations occurred with similar frequencies of 86.7 % and 83.3 % in pure and mixed LNUC, respectively. 

### 2.3. FGFR3 Immunohistochemistry

**Figure 2:**
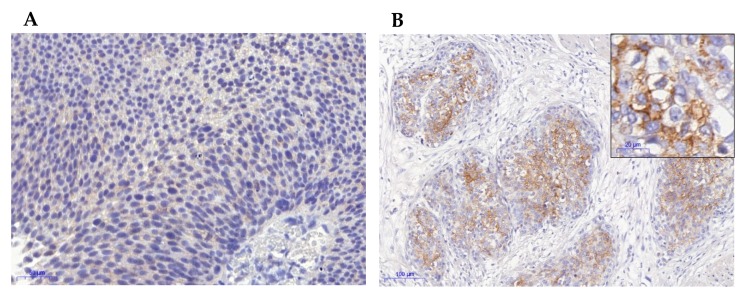
Representative images of FGFR3 immunohistochemistry: (**A**) FGFR3-negative case (Score 1); (**B**) FGFR3-positive case (score 3); (100 fold original magnification; inset: 400 fold magnification).

Immunohistochemical analysis was evaluable in 24/25 cases and revealed one (4.2%) case with score 0, nine (37.5%) with score 1, nine (37.5%) with score 2 and five (20.8%) with score 3. Figure 2 shows representative images of FGFR3 immunostaining. FGFR3 expression was significantly more frequent in *FGFR3* mutated cases (13/13 positive cases with an *FGFR3* mutation; *p* < 0.001). FGFR3 expression was significantly more frequent in pure LNUC compared to mixed cases (14/14 positive cases were pure LNUC; *p* < 0.001).

### 2.4. Immunohistochemistry (IHC)-Based Molecular Subtyping of LNUC

**Figure 3:**
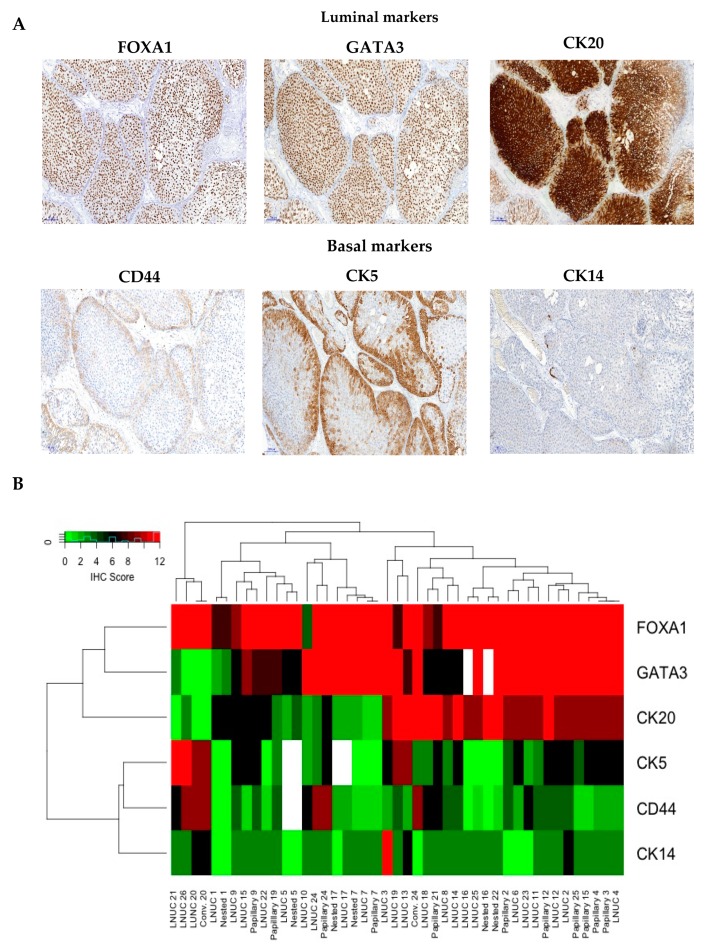
(**A**) Immunohistochemistry with luminal and basal markers, representative images (all 100 fold orginial magnification); (**B**) Heatmap presenting results of differential immunohistochemistry with luminal and basal markers; each column represents one tumor component of each patient; white fields: not available; red fields: high IRS scores; green fields: low IRS scores.

The application of a limited immunohistochemical marker panel with CK5, CK14, CD44, CK20, FOXA1 and GATA3 revealed high expression levels of luminal markers in the vast majority of LNUC cases. The differential expression of immunohistochemical markers is shown in Figure 3. Luminal markers were diffusely expressed, whereas basal markers were confined mostly to the peripheral cell layer of large nests and papillary/papillary-like structures. All the included components showed comparable marker profiles and expression levels corresponding to the LNUC area. Notably, three LNUC cases also showed a high expression of basal markers CK5 and CD44, however, histomorphological reevaluation revealed no histomorphological differences compared to the other cases.

### 2.5. PD-L1 Staining Among LNUC Cases

PD-L1 was evaluated in all components for positive tumor-associated immune cells (IC), tumor proportion score (TPS) and the combined positive score (CPS), as specified in the “Materials and Methods” section. All tumor components were stained in a similar way. The evaluation of IC showed <5% positive immune cells in 20/23 evaluable cases; evaluation of the TPS was <5% in 18/23 evaluable cases. The CPS was <10 in 19/23 evaluable cases. Table 4 displays the distribution of the percentages for the currently therapeutically relevant parameters.

## 3. Discussion

We collected one of the largest cohorts of LNUC, a rare variant of UC, which has recently been added to the nested variant of UC in the WHO classification. This is the first molecular analysis on LNUC with a focus on *FGFR3* mutations and PD-L1 expression, which are therapeutically relevant targets in UC. Furthermore, we analyzed the differential expression of six antibodies to assess the molecular subtype according to current molecular taxonomy studies. 

We found 25 urothelial carcinomas that met the histomorphological criteria of LNUC as originally described by Cox and Epstein [3]. According to their description, almost half of the cases in our cohort presented with papillary or papillary-like areas with an admixed exophytic and inverted growth pattern, referred to as a “low-grade papillary” tumor component. In addition, in our cohort LNUC was found in combination with classical nested variant and conventional urothelial carcinoma, as has been reported in the literature [3]. When LNUC was combined with a classical NVUC component, overlying carcinoma in situ was not observed, which is characteristic of the classical nested variant UC [10]. 

For 20 years, activating mutations of the *FGFR3* gene have been known as key driver mutations in approximately 70% of non-muscle invasive bladder cancers [11,12]. However, recent studies reported an approximate frequency of 12%-15% *FGFR3* mutations in MIBC [13]. This is of particular interest since, for example, the results of a phase II study investigating the *FGFR1-4* inhibitor erdafitinib reported an overall response rate of 40% (3% complete response; 37% partial response) and median progression-free survival and overall survival of 5.5 and 13.8 months, respectively, with mainly manageable side effects [14]. A phase I trial with the *FGFR1-3* inhibitor infigratinib (BGJ398) achieved an overall response rate of 25.4% and disease control rate of 64.2%, and presented estimated progression-free survival and overall survival of 3.75 and 7.75 months, respectively [15]. In our cohort, 16/17 (94.1%) pure LNUC cases presented with an activating *FGFR3* mutation, regardless of the presence of a papillary or papillary-like component. In contrast, *FGFR3* mutation analysis of mixed LNUC revealed a mutation in the LNUC component of one case only, which was LNUC combined with NVUC. This result is concordant with our recent study of an NVUC cohort, which presented *FGFR3* mutations in one of 26 (3.8%) cases [10]. This molecular diversity may also lead to the differences in the clinical behavior of pure and mixed LNUC, as reported by Compérat et al., who observed a less advanced tumor stage and a lower frequency of nodal metastasis in pure compared to mixed LNUC [5]. To sum up, the concordant findings at the clinical and molecular levels, with less aggressive behavior and *FGFR3* mutations in pure compared to mixed LNUC, indicate the possibility of different tumor entities despite the histomorphologically concordant LNUC component. As a consequence, the classification of LNUC as a separate variant of UC, rather than lumping it together with NVUC in the WHO classification, should be considered. The recognition of further components in LNUC seems to be of clinical importance and may guide the choice of therapy. 

*TERT*-promoter mutations are the most common mutations identified in UC and are independent of pathological characteristics such as grade and stage [16]. We found *TERT*-promoter mutations with similar frequencies in LNUC compared to conventional carcinomas, with percentages up to 86.7%. This finding underlines the fact that *TERT*-promoter mutations are common events in bladder cancer development [16]. Moreover, in a recently reported study of inverted and exophytic urothelial papillomas, no *TERT*-promoter mutations were identified in 11 cases [17]. Thus, *TERT*-promoter mutational analysis could be an additional tool in LNUC cases to guide pathologists as well as clinicians in diagnosis and management, especially in early disease with an inverted and papillary-like growth pattern without evident invasion of the detrusor muscle. 

Recent advances in the molecular taxonomy of UC have led to molecular subtypes, with implications for the treatment of each of those subtypes. In the most recent consensus classification system by Kamoun et al., six molecular classes were proposed, among them the luminal papillary class which includes apparently uninflamed tumors with frequent papillary growth patterns and 40% *FGFR3* mutations [9]. With an immunohistochemical marker panel limited to six antibodies, we were able to classify LNUC within the luminal subtype of UC regardless of the respective tumor component. Moreover, LNUC samples showed an *FGFR3* mutation frequency much higher than in conventional MIBC and points to the possibility of an *FGFR3*-driven scenario in pure LNUC cases. Only three tumors showed additional expression of basal markers, which would be most appropriately classified as “Urobasal B subtype” tumors of the Lund classification [9]. 

Immunotherapies have recently started to play an important role in the treatment of urothelial carcinoma [18,19]. In our study, except for four cases, all LNUC were PD-L1-negative with very low TPS and IC percentages and CPSs, which did not reach the required FDA/EMA defined cut-off levels for anti-PD1 or anti-PD-L1 monoclonal antibody treatment for patients with metastatic disease deemed ineligible for cisplatin-based chemotherapy. In one of the PD-L1 positive cases (case 7), expression levels reached IC 3% and CPS 20, thus reaching the cut-off level for CPS but not IC. According to our analysis, the majority of LNUCs do not seem to be eligible for immunotherapy. This is supported by the fact that LNUC represents the molecular subtype of luminal papillary bladder cancers, in which *FGFR3* alterations are among the most important oncogenic mechanisms [9]. The *FGFR3* oncogenic pathway is associated with a non-T-cell-inflamed cancer phenotype, characterized by reduced CD8+ T cells, chemokines, and interferons, resulting in resistance to immune checkpoint inhibitors [20]. However, combination therapies of *FGFR* inhibitors with immune checkpoint inhibitors give hope for enhanced therapeutic effects, by the modulation of the tumor micro-environment, inducing a T-cell-inflamed phenotype, which may result in a response to immune checkpoint inhibitors [20,21]. The first results regarding combination therapies were presented from a phase II study on the human anti-*FGFR3* monoclonal antibody vofatamab in combination with pembrolizumab, with responses in both *FGFR3* wild type and *FGFR3*-mutated patients, and an overall response rate of 36% [21]. Further trials with *FGFR-* and immune checkpoint inhibitor combination therapies are currently recruiting [21].

The limitations of our study are the retrospective character of our analyses and, in a subset of cases, the restricted number of tumor blocks from the limited archival tumor tissue of cases sent for consultation from other pathology departments. In those cases, we cannot completely exclude a sampling error regarding additional tumor components. In addition, mutational analysis of the investigated genes was restricted to the most common mutational hotspot regions of the *FGFR3* and the *TERT*-promoter genes and *FGFR3* rearrangement was not analyzed. The mutation hotspots analyzed in our study should cover more than 90% of bladder-cancer-relevant gain-of-function mutations of the *FGFR3* gene [13] and ~80% of *TERT* promoter mutations [22], respectively. Thus, we could have missed rare mutations by the method used in this study.

Moreover, a subclassification into basal and luminal tumors based on IHC is possible, but a detailed analysis of the luminal subtype would require an RNA-based analysis, which may be the subject of subsequent studies.

## 4. Materials and Methods 

### 4.1. Study Cohort

Twenty-five LNUCs were collected from several collaborating institutes of pathology. H&E sections from each case were reevaluated histomorphologically by (at least) two experienced pathologists (A.H., S.B., E.C.) according to the 2016 World Health Organization (WHO) Classification system [4]. The clinical and morphological characteristics of the analyzed cohort are summarized in Table 1. Twelve of 25 LNUCs were part of the cohort reported in the clinicopathological analysis published in 2017 by Compérat et al. [5]. Ethical approval for formalin-fixed and paraffin-embedded tissue was obtained from the University of Erlangen-Nürnberg. 

### 4.2. DNA Isolation

The manual microdissection of tumor tissue was performed carefully after previous annotation of the respective included components (LNUC, nested, papillary/papillary-like and conventional components). At least 80% purity of the respective component was achieved. DNA isolation was performed using the DNA preparation kit (Maxwell^®^ 16 System, Promega, Mannheim, Germany) according to the manufacturer’s instructions. Mutation analysis was performed separately for each of the tumor components.

### 4.3. FGFR3 Mutational Analysis

*FGFR3* mutational analysis was performed using the SNaPshot method, which has been described elsewhere [23]. In brief, three regions of the *FGFR3* gene (exon 7, 10 and 15), comprising nine mutations found in UC, were amplified simultaneously in a multiplex polymerase chain reaction (PCR). Seven SNaPshot primers detecting the *FGFR3* mutations p.S249C, p.R248C, p.G372C, p.Y375C, p.A393E, p.K652E, p.K652Q, p.K652M and p.K652T were annealed to the PCR products and extended with a labelled dideoxynucleotide. An automatic sequencer (ABI Prism 3500) analyzed the extended primers.

### 4.4. TERT Promoter Gene Analysis 

Mutation analysis of the *TERT* promoter was performed with SNaPshot analysis of the *TERT* core promoter with an ABI Prism 3500 Genetic Analyzer and the SNaPshot-Multiplex-Kit (Applied Biosystems, Foster City, CA, USA). The analysis was performed according to the manufacturer’s instructions. SNaPshot assays designed to detect hotspot mutations at positions -146, -124 and -57 bp of the *TERT* promoter were used. Detailed information on the method, including all primers and reaction conditions, have been described elsewhere in detail [22,24]. 

### 4.5. Immunohistochemistry

Detailed information on the antibodies used for immunohistochemical analysis are shown in Table 5. Whole slides of the tumor block were evaluated and stained following the manufacturers protocols in IHC laboratories accredited by the German Accreditation Office (DAKKs) according to DIN EN ISO/IEC 17020. A limited marker panel was chosen following the current molecular taxonomy proposals with CK5, FOXA1 and GATA3 for discrimination between luminal and basal subgroups [25]. CK5, CK14 and CD44 were previously identified and used as so-called “basal markers”; CK20 is predominately represented among luminal tumors [25,26]. Apart from a few exceptions (see below), IHC was performed on a BenchMark *ULTRA* Automated IHC/ISH Slide Staining System (Ventana Medical Systems, Tucson, AZ, USA). Manual staining was performed for FOXA1 (polyclonal, 1:1000; Abcam, ab23738). Immunohistochemical markers were analyzed according to the immunoreactive score (IRS) by Remmele and Stegner resulting from multiplication of a percentage score (0 = 0, 1 = <10%, 2 = 10–50%, 3= 51–80%, 4 = >80%) and an intensity score (assessment of the staining intensity of positive cells: 0 = negative, 1 = weak, 2 = intermediate, 3 = strong) [27]. For PD-L1 IHC, whole sections were stained and scored according to the current assay recommendations [18,19] including the immune score (IC), tumor score (TPS) and combined positive score (CPS) using the official cut-off levels (IC ≥ 5% and CPS ≥ 10) of current FDA recommendations for treatment with checkpoint inhibitors. FGFR3 IHC performed in the laboratory of one of the cooperating departments was assessed using the established semi-quantitative scoring system according to Tomlinson et al.: score 0, all tumor cells negative; score 1, faint but detectable positivity in some or all cells; score 2, weak but extensive positivity; score 3, strong positivity (regardless of extent) [28]. For statistical analysis, cases with scores 0-1 were considered negative and cases with scores 2–3 were considered positive [28].

### 4.6. Statistical Analysis 

For the analysis of nominal parameters, cross-tabulations (chi-square test) were performed with SPSS for Windows (IBM Statistics, Version 24.0, Ehningen, Germany). In the case of expected values <5 in 2 × 2 cross tabulations, a two-sided Fisher’s exact test was chosen. Results were regarded as statistically significant if *p*-values were < 0.05.

Furthermore, the R environment (R, version 3.2.3, The R Foundation for Statistical Computing, Vienna, Austria [29]) and the heatmap.2 function for non-hierarchical clustering were used. 

## 5. Conclusions

We present the first analysis of clinically relevant molecular alterations in LNUC, including pure and mixed tumors combined with other UC components. Our analysis revealed that pure LNUC may be the prototype of a luminal–papillary *FGFR3*-mutated muscle-invasive UC with very limited immune cell infiltration, mostly lacking PD-L1 expression. In contrast, *FGFR3* mutations seem to be rare in mixed LNUC cases, which indicates a different molecular background of pure versus mixed LNUC, thus the classification of LNUC as a separate variant of UC should be considered. Our results also present a potential therapeutic option in LNUC. Our findings underline the value of histomorphological examination and detailed pathology reporting, which can guide molecular analysis and the choice of specific therapies.

## Figures and Tables

**Table 1:** Clinical and morphological characteristics.

Characteristic	n (%)
**Total number of cases**	25
**Gender**	
Male	18 (81.8)
Female	4 (18.2)
Not available	3
**Stage distribution**	
pT2	15 (60.0)
pT3	7 (28.0)
pT4	2 (8.0)
pTx *	1 (4.0)
**Grade distribution (WHO 1973)**	
G2	11 (44.0)
G3	15 (56.0)
**Grade distribution (WHO 2016)**	
High-grade	25 (100)
**Histomorphology**	
Pure	17 (68.0)
Mixed	8 (32.0)
- Classical nested component	7
- Conventional component	1

WHO: World Health Organization; * pTxM1.

**Table 2:** *FGFR*3 mutations in different components of LNUC samples.

Diagnosis	LNUC	NVUC	Papillary-Like	cUC	Study Number
Pure LNUC	p.Y375C		p.Y375C		2
	p.Y375C		p.Y375C		3
	p.Y375C		p.Y375C		4
	p.R248C		p.R248C		12
	p.S249C		p.S249C		15
	p.Y375C		p.Y375C		19
	p.Y375C		p.Y375C		25
	p.Y375C		NA		9
	NA		p.S249C		21
	p.S249C				6
	p.R248C				8
	p.S249C				10
	p.S249C				11
	p.R248C				13
	p.S249C				14
	p.S249C				26
	NA				18
LNUC &	p.S249C	NA	NA		23
NVUC	WT	WT			1
	WT	WT			5
	WT	WT			16
	WT	WT			22
	WT	WT	WT		7
	NA	NA			17
LNUC & cUC	WT		WT	NA	24

WT = wild type; NA = not available.

**Table 3:** TERT-promoter mutations and the different tumor components among LNUC tumors.

Diagnosis	LNUC	Nested	Papillary-Like	Conventional	Study Number
Pure LNUC	-146 G>A		-146 G>A		25
	-124 G>A		-124 G>A /-146 G>A		12
	-124 G>A		-124 G>A		19
	-124 G>A		-124 G>A		21
	WT		-124 G>A		2
	-124 G>A		NA		9
	-57 A>C		NA		15
	-146 G>A				6
	-124 G>A				11
	-124 G>A				13
	-124 G>A				14
	-124 G>A				26
	-57 A>C				8
	WT		WT		3
	WT		WT		4
	NA				10
	NA				18
LNUC &	-124 G>A	-124 G>A	-124 G>A		7
NVUC	-124 G>A	-124 G>A			1
	-124 G>A	NA	NA		23
	WT	-146 G>A			5
	WT*	WT			16
	NA	NA			17
	NA	WT			22
LNUC &	-124 G>A		-124 G>A	-124 G>A	24
cUC					

WT = wild type; NA = not available; * −57 NA.

**Table 4:** Immunohistochemical evaluation of PD-L1 status (cut-off levels set according to current FDA/EMA recommendations).

Score	All Components n (%)
**IC (%)**	
<5%	20 (87.0)
≥5	3 (13.0)
Not available	2
**CPS**	
<10	19 (82.6)
≥ 10	4 (17.4)
Not available	2

IC: immune score, CPS: combined positive score.

**Table 5:** Antibodies used for immunohistochemistry.

Antibody	Company	Clone	Dilution
CD44	Dako	DF1485	1:40
CK20	Dako	Ks20.8	1:50
CK5	Zytomed	XM26	1:50
CK14	Ventana	SP53	ready to use
GATA3	DCS	L50-823	1:1000
FOXA1	Abcam	ab23738	1:1000
PD-L1	Dako	28-8	1:200
FGFR3	Santa Cruz	B9	1:50
